# Evaluating interventions to improve test, treat, and track (T3) malaria strategy among over-the-counter medicine sellers (OTCMS) in some rural communities of Fanteakwa North district, Ghana: study protocol for a cluster randomized controlled trial

**DOI:** 10.1186/s13063-020-04509-6

**Published:** 2020-07-08

**Authors:** Olajoju Temidayo Soniran, Benjamin Abuaku, Collins Stephen Ahorlu

**Affiliations:** 1 Noguchi Memorial Institute for Medical Research, College of Health Sciences, University of Ghana, Legon, Accra, Ghana; 2 Akanu Ibiam Federal Polytechnic, Unwana, Ebonyi State Nigeria

**Keywords:** T3 malaria strategy, mRDT, Over-the-counter medicine sellers, Interventions

## Abstract

**Background:**

The World Health Organization initiated test, treat, and track (T3) malaria strategy to support malaria-endemic countries in their efforts to achieve universal coverage with diagnostic testing, antimalarial treatment, and strengthening surveillance systems. Unfortunately, T3 is not adopted by over-the-counter medicine sellers (OTCMS) where many patients with malaria-like symptoms first seek treatment. Sub-Saharan African countries are considering introducing and scaling up RDTs in these outlets to reduce malaria burden. In this context, this study is aimed at improving implementation of the T3 among OTCMS using a number of intervention tools that could be scaled-up easily at the national level.

**Methods/design:**

The interventions will be evaluated using a two-arm, cluster randomized trial across 8 rural communities (4 clusters per arm), in two adjacent districts (Fanteakwa North and Fanteakwa South districts) of Ghana. A total of 8 OTCMS in the intervention arm and 5 OTCMS in the control arm in the selected communities will participate in the study. In the intervention arm only, subsidized malaria rapid diagnostic test (mRDT) kits will be introduced after the OTCMS have been trained on how to use the kit appropriately. Supervision, technical assistance, feedbacks, and collection of data will be provided on a regular basis at the participating medicine stores. The primary outcome is the proportion of children under 10 years with fever or suspected to have malaria visiting OTCMS and tested (using mRDT) before treatment. Secondary outcomes will include adherence to national malaria treatment guidelines and recommended mRDT retail price. Outcomes will be measured using mainly a household survey supplemented by mystery client survey and a surveillance register on malaria tests conducted by the OTCMS during patient consultations. Data collected will be double entered and verified using Microsoft Access 2010 (Microsoft Inc., Redmond, Washington) and analyzed using STATA version 11.0.

**Discussion:**

The trial will provide evidence on the combined effectiveness of provider and community interventions in improving adherence to the T3 initiative among OTCMS in rural Ghana.

**Ethical clearance:**

NMIMR-IRB CPN 086/18-19

**Trial registration:**

ISRCTN registry ISRCTN77836926. Registered on 4 November 2019.

## Background

Malaria has been managed over the years using two approaches: presumptive and test-based [1]. Presumptive treatment of malaria was practiced for decades before the World Health Organization (WHO) approved a shift in policy [2, 3]. Presently, WHO recommends treatment based on clinical grounds only if diagnostic testing is unavailable within 2 h of patients presenting for treatment, and this is necessary to effect cure and prevent life-threatening complications [4].

Prompt and accurate diagnosis of malaria is important for both rapid and effective disease management and surveillance [5]. Malaria diagnosis involves identifying malaria parasites or antigens/products in a patient’s blood [6]. Three diagnostic tools are currently in use to detect malaria parasites in infected humans: light microscopy; nucleic acid amplification (NAA) tests, most often polymerase chain reaction (PCR) based; and rapid diagnostic test (RDT) kits. Each of these tools has advantages and disadvantages. Light microscopy (often called the “gold standard” and the oldest laboratory method) detects malaria parasite species and developmental stages, can be used to quantify parasitemia, and is preferable in investigating malaria treatment failures [5, 7]. PCR detects drug-resistant parasites and may be automated to process large samples, but foremost of its advantage is higher sensitivity and specificity, that is, detects low-density malaria infections [8–10]. With respect to limitations, both light microscopy and PCR are labor intensive, are time consuming, and require trained technicians for reliable results [6]. WHO’s quest for a simple, fast, accurate, and cost-effective diagnostic test for determining the presence of malaria parasites, especially in field situations, led to the invention of malaria rapid diagnostic test (mRDT) kits [11].

mRDT kit is a device that detects specific *Plasmodium* antigen in a small quantity of fresh blood using lateral flow immunochromatography [12]. The main advantages of mRDT kits are that they require no capital investment or electricity, are simple to perform, and are easy to interpret [13]. In sub-Saharan Africa, the deployment of mRDT has increased over the past decade with much focus on the public health sector. Unfortunately, the availability and use of diagnostic testing is low among private medicine retail outlets, including pharmacies and over-the-counter medicine sellers (OTCMS), where many patients with malaria-like symptoms seek treatment [14]. Given the importance of private medicine retail sellers as a first point of care and antimalarial treatment, several endemic countries in sub-Saharan Africa and Southeast Asia are considering introducing and scaling up the use of mRDTs in these outlets to achieve universal access to prompt parasite-based diagnosis before treatment [15].

OTCMS are usually the first point of call for patients in Ghana because there are no consultation fees, little or no waiting times, and patients’ preference for self-medication. OTCMS typically operate in rural areas and are limited by the Pharmacy Act of Ghana to selling only class C (over the counter) medicines which includes antimalarial drugs. As of 2012, there were over 13,000 licensed OTCMS in Ghana out of which 233 had been accredited by the National Health Insurance Authority [16].

Studies on perception of community members and OTCMS in Ghana on proposed implementation and scale up of mRDT in malaria diagnosis reported between 67 and 97% willingness to use mRDT [17, 18]. Ansah et al., in a study conducted in Ghana, reported 100% uptake of RDT, 97% of those testing negative not receiving ACT or antimalarial medicine, and 99% of those testing positive receiving ACT, but data collection was based on provider records only [19]. Similar studies across sub-Saharan Africa using “mystery shopper” and “exit interview” as methods of collecting data recorded widely varied uptake of RDT from 8 to 88% [14]. Despite the encouraging report of mRDT uptake which is above average in Ghana, adherence to mRDT-negative test result is very low. Studies have shown that 62.0% of mRDT-negative clients were given artemether-lumefantrine, and about 30% of OTCMS and some community members held the view that mRDT-negative results did not mean no malaria illness and would therefore use ACT [17, 20].

Provision of artemisinin-based combination therapies (ACTs) and other antimalarials without a confirmed malaria test frequently results in overtreatment of malaria and reduces the availability of ACTs for true malaria cases and potentially delays the diagnosis and treatment of other causes of illness [14]. Hence, this study is aimed at improving implementation of the T3 (test, treat, and track) strategy in the informal healthcare sector [15] using a number of intervention tools that could be scaled-up easily, unlike other studies, at the national level. This will help achieve universal access to prompt parasite-based diagnosis, reduce overprescribing of antimalarial to malaria-negative clients, and promote the tracking of malaria cases.

## Methods/design

### Study design

The proposed interventions will be evaluated using a two-arm (intervention and control), cluster randomized trial across 8 rural clusters (4 clusters per arm), in two adjacent districts of Ghana. The method of Wiseman et al. will be used in this study with some modifications [21]. Wiseman et al. used a three-arm stratified cluster randomized trial to evaluate the effectiveness and cost-effectiveness of two interventions designed to support the roll-out of mRDTs and improve the rational use of ACTs. They focused in part on private medicine retail outlets (known as OTCMS in Ghana), who are the focus of this study. Wiseman et al. looked at the effectiveness of individual interventions, but this study will evaluate the combined effectiveness of different interventions.

A cluster is defined as a geographical community which contains at least one OTCMS, and this will be the unit of randomization within each study arm.

**Fig. 1:**
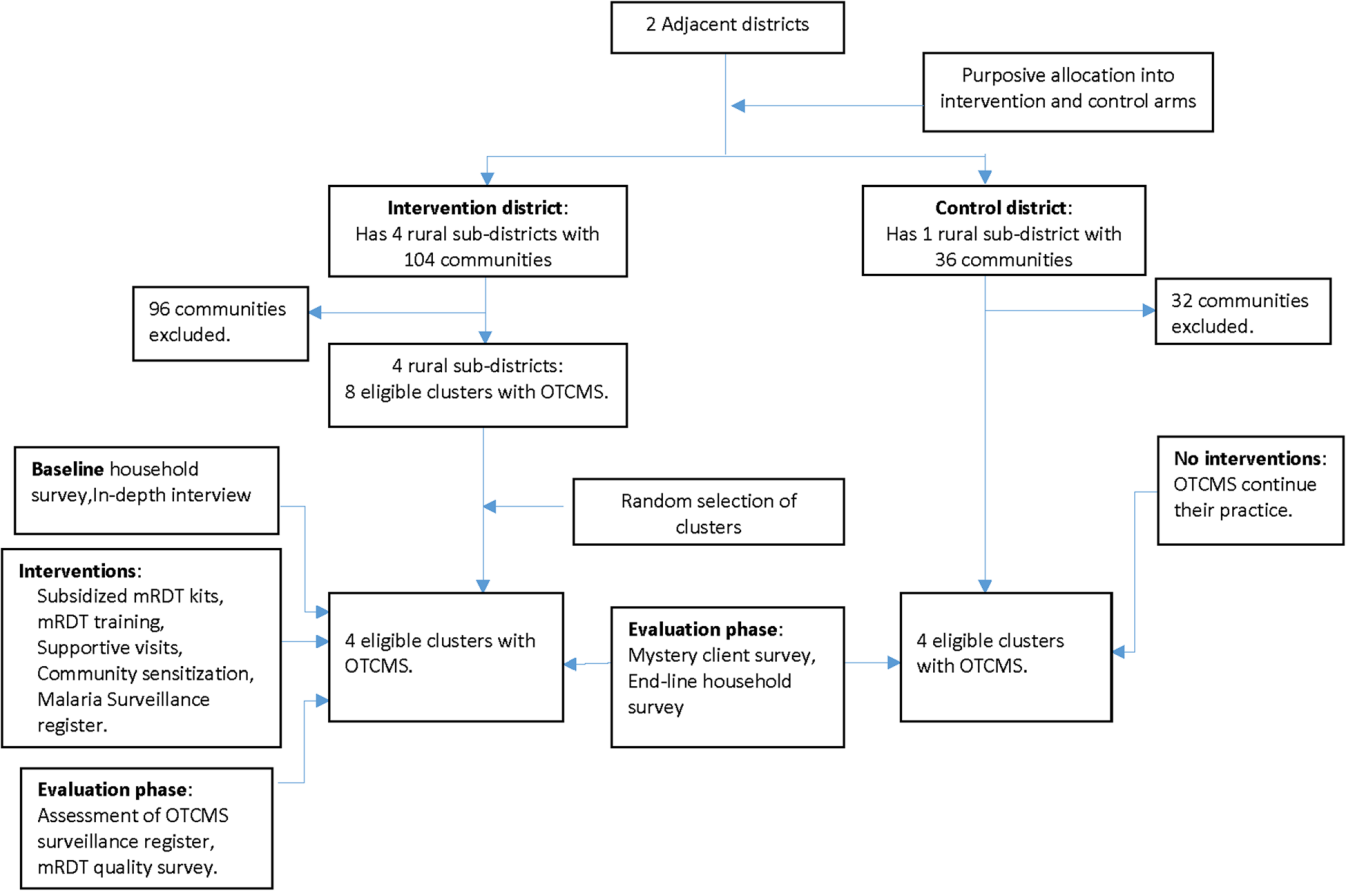
Flow chart on the study design

The intervention arm has 8 clusters, and out of these, 4 will be randomly selected using a computer-generated list. The control arm has 4 clusters, and all these will be included in the study (Fig. 1). An urban sub-district (Begoro) in the intervention district will act as a buffer between the two arms. This method is adopted to reduce contamination (that is, filtering of information across the two arms) to the barest minimum. The selected four clusters per study arm will yield a total of 8 OTCMS in the intervention arm and 5 OTCMS in the control arm.

The primary objective of the study is to evaluate the combined effectiveness of provider and community interventions by comparing malaria RDT testing rates between the intervention and control arms. The secondary objectives include:

(i) To reduce the proportion of children under 10 years who receive treatment for malaria without testing by over-the-counter medicine sellers (OTCMS) following implementation of different levels of intervention.
(ii) To determine the level of service provider (OTCMS) adherence to malaria case management guidelines.

The standard protocol items: Recommendations for intervention trials (SPIRIT 2013) checklist is presented as Additional file 1.

### Study area

**Fig. 2:**
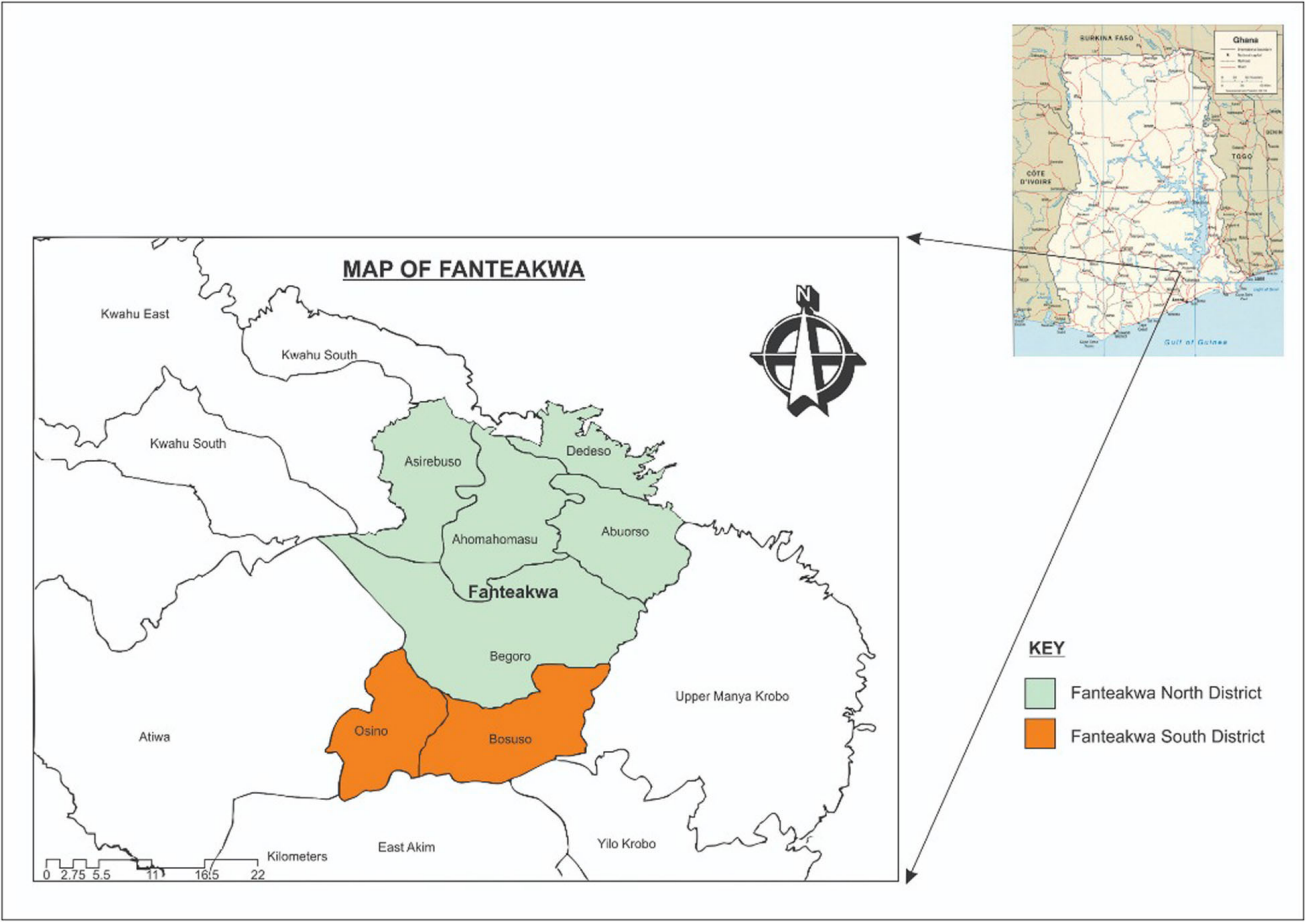
Map showing the Fanteakwa North and South districts (formerly one district—Fanteakwa)

The study will be conducted in the Fanteakwa North and Fanteakwa South districts (previously one district) in the eastern region of Ghana (Fig. 2). The capital of Fanteakwa North district is Begoro (acting as buffer) while the capital of Fanteakwa South district is Osino. The two districts cover an area of 1150 km^2^ with resident population of approximately 108,614 as of 2010. The Fanteakwa North has 5 sub-districts (one urban and four rural) while Fanteakwa South has 2 sub-districts (one urban and one rural). The two districts are characterized by double maxima rainfall in March to October, and November to February respectively, with the highest rainfall in June. They have an annual average temperature of 24 °C. The population is made up of subsistence farmers involved predominantly in crop farming and livestock rearing. The Fanteakwa North district has 1 hospital, no health center, 1 clinic, 31 community health-based planning services (CHPS) compounds, and 28 OTCMS, while the Fanteakwa South has no hospital, 2 health centers, 1 clinic, 15 CHPS compounds, and 19 OTCMS [22].

### Study procedures

The study has 4 phases. These are preparatory, baseline, intervention, and evaluation (Table 1).

**Table 1:**
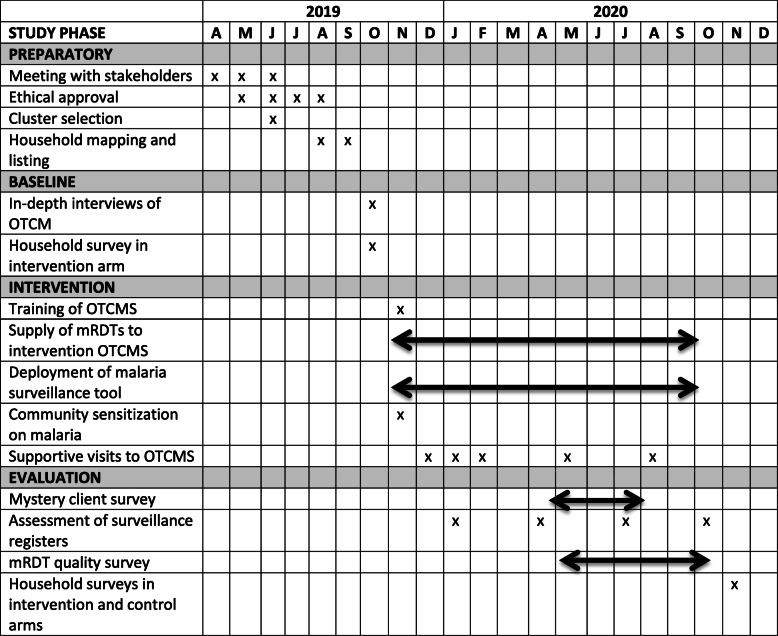
Timelines for study procedures

#### Preparatory phase

The preparatory phase will involve scheduled meetings with stakeholders including the district and regional health directorates, the National Malaria Control Programme (NMCP), relevant non-governmental organizations such as Strengthening Health Outcomes through the Private Sector (SHOPS), and OTCMS and traditional leaders in the selected communities. These engagements will create the required awareness and subsequently obtain permissions and support in the conduct of the study.

As part of the preparatory phase, houses in the selected clusters will be mapped, and lists of households with children under 10 years old will be generated with corresponding GPS coordinates.

#### Baseline phase

The baseline phase will involve conducting baseline household surveys in each of the intervention clusters and in-depth interviews of OTCMS in both the intervention and control clusters.

##### Household survey:

A household survey will be conducted in the intervention arm to document pre-intervention malaria testing rates among children under 10 years visiting OTCMS for malaria treatment in the past 1 month preceding the survey. The household questionnaire will be completed by one individual per household, usually the primary caregiver. The questionnaire will contain modules on:

- Background information and consent
- Details of the respondent
- Household members
- Treatment seeking of children under 10 years with a fever in the past 1 month preceding the survey
- Practices of OTCMS visited by caregivers with children under 10 years with a fever (i.e., malaria test done, test results, and treatment given)
- Malaria preventive measures by caregivers

##### In-depth interviews:

In-depth interviews with OTCMS in the selected clusters in both study arms will be conducted to determine possible factors preventing the effective management of malaria at their level. Their knowledge on the following themes will be explored:

- Symptoms of uncomplicated and severe malaria
- Test, treat, and track (T3) malaria initiative
- Any previous skills on the use of mRDTs
- Challenges in managing malaria at their level

In-depth interviews with OTCMS in the intervention and control arms will be held at different times by the research team and recorded for reference purposes.

#### Intervention phase

Intervention activities for this study will be (i) facilitating acquisition of subsidized mRDT kits; (ii) training OTCMS on malaria diagnosis, treatment, and tracking of cases; (iii) quarterly supportive visits to OTCMS after training; (iv) community sensitization on malaria focusing on the T3 strategy; and (v) introduction of malaria surveillance tool for use by OTCMS.

##### Acquisition of subsidized mRDT kits:

The research team will facilitate the acquisition of subsidized mRDT kits from the National Malaria Control Programme. The mRDTs will be provided to all OTCMSs in the selected intervention clusters only. mRDTs will be supplied on monthly basis to the various OTCMS participating in the study based on inventory. The OTCMS will be able to contact the research team anytime they are about to run out of stock. Stock management records will be kept by the research team to monitor the distribution of mRDTs. For the sake of identification purposes, the mRDTs will be stamped and OTCMS will be advised to store them in a cool, dry place. mRDT kits will be provided at no cost to participating OTCMS. They will be asked to price the mRDTs at a cost not more than the market wholesale price of GH¢2.40/kit (~$0.44).

##### Training of OTCMS:

Added to the supply of mRDTs, OTCMS in the selected intervention clusters will receive provider training on malaria diagnosis, treatment, and follow-ups on their clients. This additional training will be conducted over 2 days and will contain six training modules on:

1. Knowledge on malaria
2. Overview of national guidelines on malaria diagnosis, treatment, and prevention
3. Appropriate treatment when test is positive
4. Appropriate actions and treatment when test is negative
5. Effective communication
6. Importance of good-quality data in malaria surveillance: this module will discuss how the informal private healthcare sector, by keeping accurate data, can contribute meaningfully to an efficient malaria surveillance system (see additional file for sample of surveillance tool).

##### Supportive visits to OTCMS after training:

In the intervention arm, support visits will be provided to each participating OTCMS during the implementation phase to monitor and assess what the providers are doing and to reinforce the skills acquired by providers during the earlier training workshop. During the support visits, where possible, investigators will observe providers attending to patients seeking treatment for fever. Questions on the different aspects of the training will also be asked. Based on their responses, guidance will be provided on areas where the provider is experiencing difficulty. Providers will be asked about possible challenges they are experiencing on implementing what they were taught during the training. mRDT kits will also be supplied during these visits and stock of mRDT recorded. The frequency of the visit will be once a month for the first quarter (3 months) of the deployment of intervention, and afterwards quarterly (every 3 months).

##### Community sensitization on malaria:

Public health education programs on malaria (cause, diagnosis, treatment, and prevention) according to the policies of the National Malaria Control Programme and Ghana Health Services will be carried out in all clusters of the intervention arm only. Community health workers will be used to carry out this health education in churches, mosques, and schools. Communities will be sensitized on the importance of demanding malaria testing before treatment. In addition to the community health workers, community town criers will also provide sensitization on market days.

##### Introduction of malaria surveillance tool:

A malaria surveillance tool (see additional file 2) will be introduced to OTCMS at the initial training on how to use mRDTs. They will be enlightened on the importance of the tool and also trained on how to keep accurate record of all suspected malaria cases attended to using the tool. At quarterly visits, OTCMS will be engaged to elucidate possible challenges and bottlenecks experienced on the use of this tool. The community will also be sensitized on the surveillance tool.

#### Evaluation phase

The evaluation phase will include mystery client surveys, assessment of OTCMS surveillance register, mRDT quality survey, and end-line household survey.

##### Mystery client survey:

Mystery client surveys will be used to support the evaluation of OTCMS conduct in implementing the T3 strategy. Mystery client (one who pretends to be a real patient) data collection will commence in the 4th month of the intervention period. The mystery client would also assess the quality of RDT use in the intervention arm. A total of 13 mystery clients will be recruited and given intensive training over 3 days on the clinical scenario and how to fill the assessment checklist/form (see additional file 3). Practical sessions will be included. Each OTCMS will be visited twice a month by a different mystery client for 3 months during the peak malaria transmission season (May–July).

The mystery client will present at the OTCMS’s shops saying that he/she had fever that he/she thought was like a malaria fever that he/she had experienced on a previous occasion. He/she will then observe the provider’s response whether RDT will be proposed or not before treatment. The outcome of interest for the mystery client assessment will comprise 6 key steps in conducting and interpreting an RDT for malaria:

1. Provider (OTCMS) proposes to do mRDT test.
2. Use antiseptic in preparation for finger prick (to reduce the risk of infection, the mystery client will tactfully demand for the use of an antiseptic to prepare the finger before pricking if the provider does not do that).
3. Read the result correctly (providers who accurately read a negative test, assuming that all mystery clients did not have malaria).
4. Show the result to the client.
5. Provide correct treatment according to the result (i.e., providers prescribe ACT for those who read a positive test result; refrained from giving any antimalarial drugs for a negative client).
6. Cost of mRDT.

##### Assessment of OTCMS surveillance registers:

The OTCMS surveillance registers will be assessed for:

1. Symptoms warranting testing
2. Proportion of suspected malaria cases tested
3. Proportion of mRDT-negative cases given an ACT

##### mRDT quality survey:

As part of the evaluation phase, an mRDT quality survey will be conducted among suspected malaria patients visiting OTCMS in the selected intervention clusters during the peak malaria transmission season (May–October). Thick and thin blood smears for malaria microscopy will be prepared for eligible suspected uncomplicated malaria cases attended to by OTCMS. The microscopy results will be matched against mRDT results to determine the sensitivity, specificity, and predictive values of the mRDTs used.

##### Household survey:

The primary outcome will be measured through end-line household surveys. These will follow details of the baseline survey and will be conducted in both intervention and control arms.

### Sample size estimation

#### Mystery client survey

A total of 13 mystery clients will be recruited and trained. Each OTCMS will be visited twice a month by a different mystery client for 3 months (May–July). This will amount to a total of 48 visits in the intervention arm and 30 visits in the control arm.

#### mRDT quality survey

A minimum of 591 suspected uncomplicated malaria cases visiting OTCMS in the intervention clusters will be required for the mRDT quality survey based on an estimated sensitivity of 98.7% (unpublished national data), 1% precision, 20% non-response rate, and 95% confidence level.

#### Household survey

A total of 412 children under 10 years of age in 4 clusters per arm will be sampled to detect a minimum of 10% increase in malaria testing rate (from 10 to 20% in the intervention arm) and 10% difference in testing rate between the intervention arm (20%) and control arm (10%) after 12 months of deployment of interventions with 80% power and 5% significance level. This computation is based on the standard formula for randomized controlled trials described by Hemming and colleagues [23] and the application of a variance inflation factor (VIF) of 2.92 (based on an intracluster coefficient of 0.02 and an average cluster size of 97).

### Inclusion criteria

The inclusion criteria based on the different data collection methods are as follows:

#### In-depth interviews

- OTCMS within the selected clusters in both intervention and control arms

#### Baseline household survey

- Children less than 10 years old in the intervention arm only

#### mRDT quality survey

- Suspected uncomplicated malaria cases aged 6 months and above in the intervention arm only

#### End-line household survey

- Children less than 10 years old in both intervention and control arms

### Exclusion criteria

The exclusion criteria based on the different data collection methods are as follows:

#### In-depth interviews

- OTCMS outside the selected clusters

#### Baseline household survey

- Children above 10 years old

#### mRDT quality survey

- Children less than 6 months old
- Pregnant women
- Signs and symptoms of severe malaria

#### End-line household survey

- Children above 10 years old

### Safety considerations

Metallic waste bins will be provided to OTCMS in the intervention arm of the study for proper disposal of sharps used. RDT kits used will be harvested by the team for record purposes.

OTCMS will be trained on why clients with severe disease or those that are still feeling symptoms of illness but had been treated for malaria at the first visit should be referred to the nearest health center or the district hospital.

### Data management and analysis

The principal investigator (PI) will ensure that all implementation and evaluation activities are according to standard operating procedures. Quality assurance will include training of field workers/data collectors few days before data collection. The training will involve practical sessions. During data collection period, field supervisors will monitor study implementation through weekly visits to sites.

All data collected will be anonymized using only study numbers and stored in locked cabinets after entry into a computer with a password known only to investigators. Data will only be made available to authorized persons such as members of the institutional review board. Data will be double entered and verified using Microsoft Access 2010 (Microsoft Inc., Redmond, Washington) database, and later imported into STATA version 12.0 (STATA Corporation, College Station, Texas) for analysis. Any discrepancies will be corrected using source documents. To account for correlation within clusters, the generalized linear mixed model (GLMM) technique of analysis will be used in this study. Estimates from the logistic regression model with random effect will be used to determine the intervention effect. Data (recordings) collected during in-depth interviews will be transcribed, translated, and analyzed using NVivo 12 by QSR International. This will provide a better understanding of the quantitative observations.

### Outcomes

#### Primary outcome

The primary outcome is the proportion of children under 10 years with fever or suspected to have malaria visiting OTCMS and tested before treatment.

#### Secondary outcomes

Secondary outcomes include the following:

1. Proportion of children under 10 years who received antimalarial drugs from OTCMS without testing
2. Adherence to treatment guidelines:
  Percentage of clients/patients testing negative or not tested receiving ACT or other antimalarial drugs
  Percentage of clients/patients referred to the public health facility by OTCMS for further care
  Percentage of OTCMS who could accurately perform an RDT, interpret results, and dispose of waste
3. Adherence to RDT retail price: percentage of OTCMS who adhered to the recommended RDT retail price
4. The sensitivity, specificity, and predictive values of mRDTs used in the study

### Ethical considerations

Consent procedures:

- *In-depth interviews with OTCMS*: Written informed consent will be obtained from all OTCMS in the selected communities prior to their in-depth interviews during the baseline phase.
- *Household survey*: During the household survey, each eligible parent/caregiver in the household will be informed of details of the research orally by the trained interviewer. He/she will be advised to ask questions on areas that are not well understood. If he/she agrees to participate in the study, two consent forms (one for the participant and one for the interviewer) will be given to the individual to give his/her consent through signature or thumb print.
- *mRDT quality survey*: Written informed consent will be obtained from all eligible adult participants and parents of children less than 18 years. Additionally, children aged 12–17 years will also give their assent before being enrolled into the mRDT quality survey.

## Discussion

The goal of this cluster randomized controlled trial is to evaluate the combined effectiveness of provider and community interventions at improving the implementation of WHO’s T3 malaria initiative in the informal health sector in Ghana.

This trial is unique in 2 approaches. The first relates to the cost of mRDTs. mRDTs will be supplied to OTCMS at no cost to the operators. Also, the retail price for mRDTs will be pegged at the current market wholesale price. It is expected that this approach will ensure that individuals visiting OTCMS with suspected uncomplicated malaria will have a test done at a highly subsidized price while OTCMS benefit significantly. The second approach relates to regular monitoring of the activities of OTCMS. It is expected that the deployment of a surveillance tool and regular/quarterly support visits to the OTCMS will go a long way to improve on adherence to the T3 policy.

In designing this study, the main obstacle to overcome is how to ensure limited or no contamination within the control arm. Two measures are being taken: creating a buffer sub-district between the two arms and investigators engaging OTCMS directly rather than using third parties such as the leadership of the association of OTCMS at the district and regional levels.

If the trial shows that provider and community interventions deployed improved adherence to the T3 policy, the NMCP and related non-governmental organizations will be empowered with this evidence to effectively play their advocacy roles in support of the informal health sector, particularly OTCMS.

## Trial status

The current protocol is version 2.5 dated 17 September 2019. Recruitment started in September 2019 and will end in November 2020.

## Supplementary information

**Additional file 1.** SPIRIT 2013 Checklist: Recommended items to address in a clinical trial protocol and related documents.

**Additional file 2.** OTCMS' tool for surveillance of uncomplicated malaria cases.

**Additional file 3.** Checklist for malaria RDT use (for mystery shopping).

**Additional file 4.** Consent forms (in-depth interview, parental [household survey], adult [intervention phase], parental [intervention phase], child assent [intervention phase]).

## Data Availability

The data and materials generated from this study will be made available to the research community upon reasonable request to the corresponding author.
