# Supplementary material for: Evaluating interventions to improve test, treat, and track (T3) malaria strategy among over-the-counter medicine sellers (OTCMS) in some rural communities of Fanteakwa North district, Ghana: study protocol for a cluster randomized controlled trial
Source: Trials. 2020 Jul 8;21:623. doi: 10.1186/s13063-020-04509-6 (PMC7346649; doi:10.1186/s13063-020-04509-6)
Supplement: Supplementary file 2 — Additional file 2. OTCMS' tool for surveillance of uncomplicated malaria cases. [file 13063_2020_4509_MOESM2_ESM.docx]

**OTCMSs TOOL FOR SURVEILLANCE OF UNCOMPLICATED MALARIA CASES**

Community: ________________________

Shop number/code: ______________________

| **No.** | **Date**  **(dd/mm/yy)** | **Name** | **Sex**  **(M/F)** | **Age**  **(yrs)** | **Presenting symptoms** | **Tested (Yes/No)** | **mRDT results**  **(+ve /-ve /invalid)** | **Treatment**  **(Medicines dispensed)** | **General Remarks** |
| --- | --- | --- | --- | --- | --- | --- | --- | --- | --- |
|  |  |  |  |  |  |  |  |  |  |
|  |  |  |  |  |  |  |  |  |  |
|  |  |  |  |  |  |  |  |  |  |
|  |  |  |  |  |  |  |  |  |  |
|  |  |  |  |  |  |  |  |  |  |
|  |  |  |  |  |  |  |  |  |  |
|  |  |  |  |  |  |  |  |  |  |
|  |  |  |  |  |  |  |  |  |  |
|  |  |  |  |  |  |  |  |  |  |
|  |  |  |  |  |  |  |  |  |  |
|  |  |  |  |  |  |  |  |  |  |
|  |  |  |  |  |  |  |  |  |  |
|  |  |  |  |  |  |  |  |  |  |
