# Supplementary material for: Evaluating interventions to improve test, treat, and track (T3) malaria strategy among over-the-counter medicine sellers (OTCMS) in some rural communities of Fanteakwa North district, Ghana: study protocol for a cluster randomized controlled trial
Source: Trials. 2020 Jul 8;21:623. doi: 10.1186/s13063-020-04509-6 (PMC7346649; doi:10.1186/s13063-020-04509-6)
Supplement: Supplementary file 3 — Additional file 3. Checklist for malaria RDT use (for mystery shopping). [file 13063_2020_4509_MOESM3_ESM.docx]

| **CHECKLIST FOR MALARIA RDT USE**  **(FOR MYSTERY SHOPPING)**  **Community____________ Shop Number/Code:_________** | | | |
| --- | --- | --- | --- |
| **S/N** | **QUESTION** | **YES** | **NO** |
| 1 | Did the provider check the temperature of the client using a thermometer? |  |  |
| 2 | Did the provider check the expiry date of the RDT kit? |  |  |
| 3 | Did the provider check to see if the RDT package contained all the materials needed before performing the test? |  |  |
| 4 | Did the provider put on gloves before performing the test? |  |  |
| 5 | Was the right labeling done on the cassette? |  |  |
| 6 | Did the provider use the recommended finger to conduct the test? |  |  |
| 7 | Was the finger disinfected before pricking? |  |  |
| 8 | Was the lancet disposed of correctly? |  |  |
| 9 | Was the first blood wiped off with a cotton wool? |  |  |
| 10 | Was the right amount of blood collected? |  |  |
| 11 | Did the provider place the blood sample in the appropriate well? |  |  |
| 12 | Was the used blood-collecting device disposed of correctly? |  |  |
| 13 | Did the provider place the buffer in the appropriate well? |  |  |
| 14 | Was the recommended amount of buffer added to the test? |  |  |
| 15 | Did the provider immediately check the time after adding the buffer? |  |  |
| 16 | Did the provider dispose of the other waste materials correctly? |  |  |
| 17 | Did the provider wait for the recommended time (15-30mins) to elapse before reading the results? |  |  |
| 18 | Was the provider able to interpret the result correctly? |  |  |
| 19 | Was the provider able to complete the reporting forms correctly? |  |  |
| 20 | Does the facility have ACT in stock? |  |  |

Name of Mystery Client:________________________________________________________________

Signature of Mystery Client:_________________ Time of visit:________ (morning/afternoon/ evening)

Date of visit: ___________________________________
