# Supplementary material for: Evaluating interventions to improve test, treat, and track (T3) malaria strategy among over-the-counter medicine sellers (OTCMS) in some rural communities of Fanteakwa North district, Ghana: study protocol for a cluster randomized controlled trial
Source: Trials. 2020 Jul 8;21:623. doi: 10.1186/s13063-020-04509-6 (PMC7346649; doi:10.1186/s13063-020-04509-6)
Supplement: Supplementary file 4 — Additional file 4. Consent forms (in-depth interview, parental [household survey], adult [intervention phase], parental [intervention phase], child assent [intervention phase]). [file 13063_2020_4509_MOESM4_ESM.pdf]

NMIMR-IRB CONSENT FORM FOR IN-DEPTH INTERVIEW

Title: "IMPROVING IMPLEMENTATION OF THE TEST TREAT TRACK (T3) MALARIA STRATEGY
IN THE INFORMAL HEALTH SECTOR"

Principal Investigator: **SONIRAN OLAJOJU TEMIDAYO (Ph.D)**

Address: **NOGUCHI MEMORIAL INSTITUTE FOR MEDICAL RESEARCH, LEGON; UNIVERSITY OF GHANA, ACCRA. GHANA.**

General Information about Research
I am doing research on malaria which is very common in this community and in this region. I am going to give you information and invite you to be part of this research.
This consent form may contain words that you do not understand. Please ask me to stop as we go through the information and I will take time to explain. If you have any questions, feel free to ask me.

Purpose of the research
Malaria is making many people sick in this community especially young children and pregnant women. We want to find ways to stop this from happening. We believe that you can help us by telling us what you know about malaria, how you know when someone has malaria, how you treat malaria and difficulties you experience while treating people with malaria. All these information might help us to learn how to better control malaria in this community.

Type of Research Intervention
This research on malaria will involve your participation by answering some questions which might take about 50 minutes to 1 hour of your time.

Participant Selection
You are being invited to take part in this research because we feel that your experience as a medicine seller and care giver can contribute much to our understanding and knowledge of how malaria should be treated.

Procedures
(A). We are inviting you to take part in this research project. If you accept, you will be asked to answer some few questions.
(B). If you do not wish to answer any of the questions, kindly inform me and I will move on to the next question.
(D). You don't need to mention your name while the interview is going on. The interview will be recorded and the tapes will be stored for about 2 years (period of the study).

Possible Benefits
There will be no direct benefit to you, but your participation is likely to help us find out more about how to prevent and treat malaria in your community and this region as a whole.

Confidentiality
The information you provide is confidential, your name is not being included in the recordings, and no one else except me and Dr. Soniran O.T. will have access to your information.

Additional Cost
There is no additional cost for your participation.

Voluntary Participation and Right to Leave the Research
Your participation in this research is entirely voluntary. It is your choice whether to participate or not. If you choose not to participate, your decision will not deny you of your rights of any kind. You also have the right not to participate at any point in time.

Contacts for Additional Information
In case you have any pertinent question/questions, please feel free to call Dr. Soniran O.T. on 0269801672.

Your rights as a Participant
This research has been reviewed and approved by the Institutional Review Board of Noguchi Memorial Institute for Medical Research (NMIMR-IRB). If you have any questions about your rights as a research participant you can contact the IRB Office between the hours of 8am-5pm through the landline 0302916438 or email addresses: nirb@noguchi.ug.edu.gh

VOLUNTEER AGREEMENT

The above document describing the benefits, risks and procedures for the research title ***(***Improving Implementation of the Test Treat Track (T3) Malaria Strategy In The Informal Health Sector***)*** has been read and explained to me. I have been given an opportunity to have any questions about the research answered to my satisfaction. I agree to participate as a volunteer.

________________________ ____

Date Name and signature or mark of volunteer

If volunteers cannot read the form themselves, a witness must sign here:

I was present while the benefits, risks and procedures were read to the volunteer. All questions were answered and the volunteer has agreed to take part in the research.

________________________ ______________________________

Date Name and signature of witness

I certify that the nature and purpose, the potential benefits, and possible risks associated with participating in this research have been explained to the above individual.

________________________ ____

Date Name Signature of Person Who Obtained Consent

NMIMR-IRB PARENTAL CONSENT FORM for Household Survey

Title: "IMPROVING IMPLEMENTATION OF THE TEST TREAT TRACK (T3) MALARIA STRATEGY IN THE INFORMAL HEALTH SECTOR"

Principal Investigator: **SONIRAN OLAJOJU TEMIDAYO (Ph.D)**

Address: **NOGUCHI MEMORIAL INSTITUTE FOR MEDICAL RESEARCH, LEGON; UNIVERSITY OF GHANA, ACCRA. GHANA.**

General Information about Research
I am doing malaria research with the Noguchi Memorial Institute for Medical Research, University of Ghana, Legon. Malaria is a very common disease in this community and region affecting mostly children and pregnant women. I am inviting you to be part of this research because your house is one of the houses that have been randomly selected for the research.
This consent form may contain words that you do not understand. Please ask me to stop as we go through the information and I will take time to explain. If you have any questions, feel free to ask me.

Purpose of the research
The purpose of this study is to obtain information from caregivers of children less than 10 years old to help us learn how to better control malaria in this community and beyond. Malaria is making many people sick in this community especially young children and pregnant women. We want to find ways to stop this from happening. We believe that you can help us by telling us what you know about malaria, how you prevent malaria, how you know when someone has malaria, and how you treat malaria. All these information will help us learn how to better control malaria in this community.

Procedures
If you accept to be part of this research, you will be asked a few questions about yourself and all your children who are less than 10 years old. This will not take more than 10 minutes. You will be asked what you know about malaria, how you prevent malaria, how you know when someone has malaria, and how you treat your children when you think they have malaria.

Possible Risks and Discomfort
This research poses no direct physical harm. However, if you are uncomfortable with any of the questions I'll ask you, kindly prompt me to stop.

Possible Benefits
There will be no direct benefit to you, but your participation will contribute to the information we will be sharing with the National Malaria Control Programme on how to control malaria in this community and region as a whole.

Confidentiality
The information you provide is confidential, your name is not being included on the forms, only a number will identify you, and no one else except Dr. Soniran O.T. and two staff (Statisticians) of Noguchi Memorial Institute for Medical Research will have access to your survey.

Voluntary Participation and Right to Leave the Research
Your participation in this research is entirely voluntary. It is your choice whether to participate or not. If you choose not to participate, your decision will not deny you of your rights of any kind. You also have the right not to participate at any point in time.

Contacts for Additional Information
In case you have any pertinent question/questions, please feel free to call Dr. Soniran O.T. on 0269801672.

Your rights as a Participant
This research has been reviewed and approved by the Institutional Review Board of Noguchi Memorial Institute for Medical Research (NMIMR-IRB). If you have any questions about your rights as a research participant you can contact the IRB Office between the hours of 8am-5pm through the landline 0302916438 or email addresses: nirb@noguchi.ug.edu.gh

VOLUNTEER AGREEMENT

The above document describing the benefits, risks and procedures for the research title ***(***Improving Implementation of the Test Treat Track (T3) Malaria Strategy In The Informal Health Sector***)*** has been read and explained to me. I have been given an opportunity to have any questions about the research answered to my satisfaction. I agree to participate as a volunteer.

_________________________ ____

Date Name and signature or mark of volunteer

If volunteers cannot read the form themselves, a witness must sign here:

I was present while the benefits, risks and procedures were read to the volunteer. All questions were answered and the volunteer has agreed to take part in the research.

_________________________ ____

Date Name and signature of witness

I certify that the nature and purpose, the potential benefits, and possible risks associated with participating in this research have been explained to the above individual.

_________________________ ____

Date Name Signature of Person Who Obtained Consent

NMIMR-IRB ADULT CONSENT FORM FOR OTCMS CLIENTS (Intervention phase)

Title: "IMPROVING IMPLEMENTATION OF THE TEST TREAT TRACK (T3) MALARIA STRATEGY IN THE INFORMAL HEALTH SECTOR"

Principal Investigator: **SONIRAN OLAJOJU TEMIDAYO** (Ph.D)

Address: **NOGUCHI MEMORIAL INSTITUTE FOR MEDICAL RESEARCH, LEGON; UNIVERSITY OF GHANA, ACCRA. GHANA**

General Information about Research
I am doing malaria research with the Noguchi Memorial Institute for Medical Research. Malaria is a very common disease in this community and region affecting mostly children and pregnant women. I am going to give you information and invite you to be part of this research.
This consent form may contain words that you do not understand. Please ask me to stop as we go through the information and I will take time to explain. If you have any questions, feel free to ask me.

Purpose of the research
The purpose of this research is to determine how accurate the malaria test done in this shop is.

Procedures
If you accept to be part of this study, you will be asked to undergo a malaria test using 4 drops of blood that will be obtained from a finger prick. The first drop will be wiped off; the second drop will be used to do a quick test for the presence of malaria germs; the third and fourth drops will be placed on a small glass for examination under a microscope later. You will wait for about 25 minutes to get the result of the quick malaria test. If you test positive for malaria, the medicine seller will prescribe a drug for you. On the other hand, if you test negative, you will be referred to the nearest government approved health facility for further diagnosis on possible causes of your illness.

Possible Risks and Discomforts
You will experience little pain on the finger at the point of the prick but this will subside within 5-10 minutes. Additionally, dry sterile cotton wool will be applied to stop the bleeding from the finger prick.

Possible Benefits
There will be no direct benefit to you, but your participation will help us determine how accurate the quick test done in this shop is. This information will contribute to strategies for malaria control in this community and the country as a whole.

Confidentiality
The information you provide is confidential, your name is not being included on the forms, only a number will identify you, and no one else except Dr. Soniran O.T. and two staff (Statisticians) of Noguchi Memorial Institute for Medical Research will have access to any information about you.

Voluntary Participation and Right to Leave the Research
Your participation in this research is entirely voluntary. It is your choice whether to participate or not. If you choose not to participate, your decision will not deny you of your rights. You also have the right not to participate at any point in time.

Contacts for Additional Information
In case you have any pertinent question/questions, please feel free to call Dr. Soniran O.T. on 0269801672.

Your rights as a Participant
This research has been reviewed and approved by the Institutional Review Board of Noguchi Memorial Institute for Medical Research (NMIMR-IRB). If you have any questions about your rights as a research participant you can contact the IRB Office between the hours of 8am-5pm through the landline 0302916438 or email addresses:

VOLUNTEER AGREEMENT

The above document describing the benefits, risks and procedures for the research title ***(***Improving Implementation of the Test Treat Track (T3) Malaria Strategy In The Informal Health Sector***)*** has been read and explained to me. I have been given an opportunity to have any questions about the research answered to my satisfaction. I agree to participate as a volunteer.

________________________ ____
Date Name and signature or mark of volunteer

If volunteers cannot read the form themselves, a witness must sign here:

I was present while the benefits, risks and procedures were read to the volunteer. All questions were answered and the volunteer has agreed to take part in the research.

________________________ ____
Date Name and signature of witness

I certify that the nature and purpose, the potential benefits, and possible risks associated with participating in this research have been explained to the above individual.

________________________ ____
Date Name Signature of Person Who Obtained Consent

NMIMR-IRB PARENTAL CONSENT FORM FOR OTCMS CLIENTS (Intervention phase)

Title: 'IMPROVING IMPLEMENTATION OF THE TEST TREAT TRACK (T3) MALARIA STRATEGY IN THE INFORMAL HEALTH SECTOR.'

Principal Investigator: Dr Soniran O.T.

Address: Epidemiology Department; Noguchi Memorial Institute for Medical Research, Legon. University of Ghana, Accra. Ghana.

General Information about Research
I am doing malaria research with the Noguchi Memorial Institute for Medical Research. Malaria is a very common disease in this community and region affecting mostly children and pregnant women. We are doing this research among people (aged 6 months and above) visiting this shop with signs and symptoms that suggest they have malaria. Your child has been invited to participate in this study because he/she has signs and symptoms that suggest that he/she has malaria.

Purpose of the research
The purpose of this research is to determine how accurate the malaria test done in this shop is.

Procedures
If you accept that your child should be part of this study, he/she will be asked to undergo a malaria test using 4 drops of blood that will be obtained from a finger prick. The first drop will be wiped off; the second drop will be used to do a quick test for the presence of malaria germs; the third and fourth drops will be placed on a small glass for examination under a microscope later. You will wait for about 25 minutes to get the result of the quick malaria test. If your child tests positive for malaria, the medicine seller will prescribe a drug for him/her. On the other hand, if he/she tests negative, he/she will be referred to the nearest government approved health facility for further diagnosis on possible causes of his/her illness.

Possible Risks and Discomforts
Your child will experience little pain on the finger at the point of the prick but this will subside within 5-10 minutes. Additionally, dry sterile cotton wool will be applied to stop the bleeding from the finger prick

Possible Benefits
There will be no direct benefit to your child, but his/her participation will help us determine how accurate the quick test done in this shop is. This information will contribute to strategies for malaria control in this community and the country as a whole.

Confidentiality
The information you provide is confidential, your child's name is not being included on the forms, only a number will identify him/her, and no one else except Dr. Soniran O.T. and two staff (Statisticians) of Noguchi Memorial Institute for Medical Research will have access to any information about your child.

Voluntary Participation and Right to Leave the Research
Your child's participation in this research is entirely voluntary. If you choose not to allow him/her to participate, your decision will not deny him/her of his/her rights. You also have the right not to allow him/her to participate at any point in time.

Contacts for Additional Information
In case you have questions on the research, please call Dr Soniran O.T. on 0269801672.

Your Child's Rights as a Participant
This research has been reviewed and approved by the Noguchi Memorial Institute for Medical Research Institutional Review Board (NMIMR-IRB). If you have any questions about your child's rights as a research participant you can contact the IRB Office between the hours of 8am-5pm through the landline 0302916438 or email addresses: nirb@noguchi.ug.edu.gh

VOLUNTEER AGREEMENT

The above document describing the benefits, risks and procedures for the research title **'**Improving Implementation of the Test Treat Track (T3) Malaria Strategy In The Informal Health Sector**'** has been read and explained to me. I have been given an opportunity to have any questions about the research answered to my satisfaction. I agree that my child should participate as a volunteer.

______________________ ____
Date Name and signature or mark of parent or guardian

If volunteers cannot read the form themselves, a witness must sign here:

I was present while the benefits, risks and procedures were read to the child's parent or guardian. All questions were answered and the child's parent has agreed that his or her child should take part in the research.

______________________ ____
Date Name and signature of witness

I certify that the nature and purpose, the potential benefits, and possible risks associated with participating in this research have been explained to the above individual.

______________________ ____
Date Name Signature of Person Who Obtained Consent

NOGUCHI MEMORIAL INSTITUTE FOR MEDICAL RESEARCH INSTITUTIONAL REVIEW BOARD (NMIMR-IRB) CHILD ASSENT FORM

Introduction

I am working with the Noguchi Memorial Institute for Medical Research, University of Ghana, Legon on a research entitled "IMPROVING IMPLEMENTATION OF THE TEST TREAT TRACK (T3) MALARIA STRATEGY IN THE INFORMAL HEALTH SECTOR." I am asking you to take part in this research because the signs and symptoms you are presenting here suggests that you have malaria, and your parent(s) has/have agreed that you participate in the research. The purpose of this research is to determine how accurate the malaria test done in this shop is.

General Information

If you accept to be part of this study, you will be asked to undergo a malaria test using 4 drops of blood that will be obtained from a finger prick. The first drop will be wiped off; the second drop will be used to do a quick test for the presence of malaria germs; the third and fourth drops will be placed on a small glass for examination under a microscope later. You will wait for about 25 minutes to get the result of the quick malaria test. If you test positive for malaria, the medicine seller will prescribe a drug for you. On the other hand, if you test negative, you will be referred to the nearest government approved health facility for further diagnosis on possible causes of your illness.

Possible Benefits

There will be no direct benefit to you but your participation will help us determine how accurate the quick test done in this shop is.

Possible Risks and Discomforts

You will experience little pain on the finger at the point of the prick but this will subside within 5-10 minutes. Additionally, dry sterile cotton wool will be applied to stop the bleeding from the finger prick.

Voluntary Participation and Right to Leave the Research

Even though your parent (s) has/have agreed that you should participate in the research, you are at liberty to refuse to participate, and nobody will be angry with you. You can stop participating at any time if you feel uncomfortable.

Confidentiality

Your information will be kept confidential. No one will be able to know how you responded to the questions and your information will be anonymous.

Contacts for Additional Information

You may ask me any questions about this study. You can call me at any time (0269801672) or talk to me the next time you see me.

Your rights as a Participant

This research has been reviewed and approved by the Institutional Review Board of Noguchi Memorial Institute for Medical Research (NMIMR-IRB). If you have any questions about your rights as a research participant you can contact the IRB Office between the hours of 8am-5pm through the landline 0302916438 or email addresses: nirb@noguchi.ug.edu.gh

VOLUNTARY AGREEMENT

By making a mark or thumb printing below, it means that you understand and know the issues concerning this research study. If you do not want to participate in this study, please do not sign this assent form. You and your parents will be given a copy of this form after you have signed it.

This assent form which describes the benefits, risks and procedures for the research titled 'Improving Implementation of the Test Treat Track (T3) Malaria Strategy In The Informal Health Sector' has been read and or explained to me. I have been given an opportunity to have any questions about the research answered to my satisfaction. I agree to participate.

Child's Name: …………………………….. **Child's Mark/Thumbprint ……….………**

Date: **………………………………………..**

Researcher's Name: ………………………. **Researcher's Signature: ………………….**

Date: ……………………………………………

A witness must sign here:

I was present while the benefits, possible risks and procedures were read to the child. All questions were answered and the child has agreed that he/she will take part in the research.

________________________ ________________________________

Date Name and signature of witness
